# Orientation and Incorporation of Photosystem I in Bioelectronics Devices Enabled by Phage Display

**DOI:** 10.1002/advs.201600393

**Published:** 2017-01-31

**Authors:** Pavlo Gordiichuk, Diego Pesce, Olga E. Castañeda Ocampo, Alessio Marcozzi, Gert‐Jan A. H. Wetzelaer, Avishek Paul, Mark Loznik, Ekaterina Gloukhikh, Shachar Richter, Ryan C. Chiechi, Andreas Herrmann

**Affiliations:** ^1^ Department of Polymer Chemistry and BioengineeringZernike Institute for Advanced MaterialsUniversity of GroningenNijenborgh 49747 AGGroningenThe Netherlands; ^2^ Stratingh Institute for Chemistry and Zernike Institute for Advanced MaterialsUniversity of GroningenNijenborgh 49747 AGGroningenThe Netherlands; ^3^ The Bio and Molecular Electronics GroupDepartment of Materials Science and EngineeringFaculty of Engineering and University Center for Nano Science and NanotechnologyTel Aviv UniversityTel‐Aviv69978Israel

**Keywords:** biomimetics, biophotovoltaics, phage display, photosystem I, solar cells

## Abstract

**Interfacing proteins with electrode surfaces** is important for the field of bioelectronics. Here, a general concept based on phage display is presented to evolve small peptide binders for immobilizing and orienting large protein complexes on semiconducting substrates. Employing this method, photosystem I is incorporated into solid‐state biophotovoltaic cells.

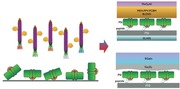

Due to the high internal efficiency of the multiprotein complex photosystem I (PSI) to perform light harvesting and charge separation, this key component of photosynthesis potentially enables biophotovoltaic devices in which the active components are biologically grown rather than chemically synthesized. However, owing to the size and structural complexity it remains a challenge to assemble well‐oriented monolayers of PSI on surfaces, which is critical for device functionality due to the directional bias of the electron‐transfer process. We applied phage‐display techniques to identify short binding peptides that afford near‐perfect control over the orientation of PSI in self‐assembled monolayers (SAMs) once these peptides are functionalized with a surface anchoring group. Almost 100% orientation of PSI on a semiconductor substrate was achieved. We characterized the orientation in junctions by using soft, conformal top contacts comprising eutectic gallium‐indium (EGaIn), and using single‐complex conducting atomic force microscopy (c‐AFM), and demonstrated functionality by fabricating solid‐state bulk heterojunction (BJH) solar cells.

Phage display (PD) is a very powerful method for the selection of peptides that bind a wide variety of molecules and substrates.^1^ Initially, it was employed to identify binders against proteins for the development of therapeutic antibodies.^2^, ^3^ Closely related applications are the selection of small‐molecule enzyme inhibitors from phage‐displayed combinatorial peptide libraries^4^, ^5^ and the generation of peptide‐dendrimer hybrids targeting the structural protein collagen.^6^ Substrates other than proteins rapidly came into the purview of PD. Small peptide binders were evolved against natural polymers such as chitin,^7^ synthetic macromolecules including isotactic poly(methyl methacrylate),^8^ and the conjugated fluorescent polymer poly(p‐phenylene vinylene).^9^ PD even enabled the identification of binders of small organic molecules, ranging from fluorescent dyes^10^ to volatile explosives such as trinitrotoluene.^11^ Besides biomolecular substrates, organic small molecule, and macromolecule targets, PD has been used to find binders against inorganic materials, for example, different allotropes of carbon including graphene^12^ and carbon nanotubes,^13^ metals such as silver,^14^ and semiconductors such as GaAs^15^ or ZnS.^16^ This utilization of the binding motifs against inorganic substrates allows the fabrication of complex multicomponent devices by displaying selected peptides on the surfaces of phages. For example, viruses with a motif binding single walled carbon nanotubes and a sequence recognizing iron phosphate act as environmentally benign low‐temperature biological template for the fabrication of state‐of‐the‐art lithium‐ion batteries.^17^ Virus particles with PD‐derived peptides can also act as a biological scaffold for the assembly of active materials for use in dye‐sensitized solar cells.^18^

A natural key building block for transforming light into chemical energy is the multiprotein complex PSI from thermophilic cyanobacteria such as *Thermosynechococcus elongatus*, which has found widespread use as a model system for structural and function studies. PSI from *T. elongatus* consists of main subunits shown in **Figure** **1**A,B: PsaA, PsaB, PsaC, PsaD, PsaE, PsaF, PsaI, PsaJ, PsaK, PsaL, PsaM, and PsaX.^19^ Dispersed within the protein scaffold are 127 cofactors: three iron‐sulfur (Fe_4_S_4_) clusters, one calcium ion, 22 β‐carotene, 96 chlorophyll a, 1,2‐dipalmitoyl‐phosphatidyl‐glycerol, one 1,2‐distearol‐monogalactosyl‐diglyceride, and two phylloquinone molecules. Under certain conditions, PSI complexes trimerize with a threefold rotation symmetry axis (C3) oriented perpendicular to the membrane (along with photogenerated charges also flow). The PsaL subunit connects the PSI monomers within the trimer configuration. PsaA and PsaB are the largest subunits containing eleven transmembrane helices. Both subunits bind a large number of chlorophyll molecules and coordinate the electron transport chain (ETC), which is lining the inside of the protein complex. The subunits PsaC, PsaD, and PsaE form a crescent shape and are located at the stroma site of the thylakoid membrane. PsaC coordinates the two terminal Fe_4_S_4_ clusters F_A_ and F_B_ of the ETC.

**Figure 1:**
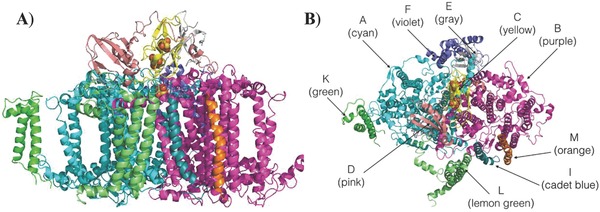
Crystal structure of PSI from A) side view and B) top view. Different protein subunits are color‐coded and the three iron‐sulfur (Fe_4_S_4_) clusters are indicated as colored atoms in yellow and orange.

Photons harvested by chlorophyll molecules are transferred to the reaction center P700, where charge separation takes place. At P700, the special pair of chlorophylls forms an excited state with a 1.3 V potential difference. From there, the electron is transferred via the primary electron acceptors A_0_ (Chla), A_1_ (phylloquinone), F_X_, F_A_, and F_B_ (Fe_4_S_4_ clusters) within the complex to the external ferredoxin charge carrier. The corresponding positive charge generated at the center of P700^+•^ remains at the opposite side of the protein complex (the lumen side of the thylakoid membrane) and is recharged by electrons from cytochrome c_6_ (and plastocyanin). The process of light harvesting and charge separation is very efficient, with an internal quantum efficiency close to unity. For that reason, PSI complexes have been incorporated into bio‐photonic devices, biosolar and biofuel cells.^20^, ^21^, ^22^, ^23^ Further improvements serving the purpose of photocurrent generation were achieved by implanting Pt nanocrystals in PSI and the fabrication of a nanocomposite by electropolymerization of thioaniline on electrode surfaces.^24^ However, the application in such devices remains limited by the confines of device architectures, where the principle challenge is overcoming the tendency of PSI to self‐assemble with random orientations on surfaces due to its structural complexity—hydrophobic sides, polar stoma, and lumen areas—as well as its relative large size (on the order of 10 nm). To solve this problem, we propose the utilization of PD technology to identify new, short binding peptides capable of directing the orientation of PSI complexes on surfaces.

In this Communication, we demonstrate a new phage display protocol to select small peptide binders against PSI trimers chemically bound to the surface of a solid substrate. From this selection procedure, we identified three linear 12 mer peptides with high affinity against the stromal side of PSI. After chemical synthesis and the introduction of a surface anchor, these peptides can bias the orientation of PSI in SAMs such that the electron accepting F_B_ cluster points directing into the substrate; one peptide produces SAMs with nearly 100% orientation. We determined the orientation by c‐AFM on single complexes and using EGaIn on the macroscale; the orientation of PSI with respect to the surface of a bottom electrode gives rise to direction‐dependent asymmetry in tunneling currents. We demonstrated the functionality of oriented SAMs of PSI by assembling them on indium tin oxide (ITO) electrodes in BJHs biophotovoltaic solar cells where the magnitude of the open‐circuit voltage (*V*_oc_) correlated to the degree of orientation.

Photosystem I from *T. elongatus* was purified as a trimer and attached to an epoxide‐modified surface (see Supporting Information), which resulted in functionalization of amino groups to cause PSI to orient randomly. Next, a commercial library of filamentous bacteriophage M13 expressing 12‐amino acid long random‐peptide sequences in fusion with their p3 protein (NEB #E8110S) was panned against the immobilized PSI trimer targets. Although the protocol of the vendor was followed, after three rounds of panning and amplification, no selection was achieved. Instead, wild‐type phages were recovered after the final amplification step.

Aiming for a reduction of the number of cycles that could favor the selection of wild‐type phages, we developed a new phage display method that leverages enzymes conjugated to the viral particles (**Figure** **2**A,B). Usually, during PD selection, phages are exposed to the target and can be eluted after washing without knowing the exact level of phages bound to the immobilized target. The new PD protocol allowed determining the amount of phages left after each wash and enabled a decision whether to continue with the washes or proceed with the elution. In our strategy, prior to panning, M13 phages were covalently functionalized with horse radish peroxidase (HRP), which allowed monitoring in real time the level of phages bound to the surface by turnover of a fluorogenic substrate. After the washing, only the PSI‐bound phages were retained in the well and we were able to quantify the amount of phages by measuring the fluorescent signal produced by the HRP. In this way, only the few phages strongly interacting with PSI were collected, enabling the generation of aptamers against PSI in a single round of selection.

**Figure 2:**
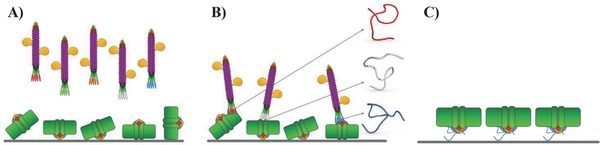
Schematic of phage display technique against PSI. A) PSI (green) with Fe_4_S_4_‐clusters (red diamonds) immobilized randomly on a surface is incubated with a library of M13 phages (magenta) covalently functionalized with horse radish peroxidase (HRP, yellow) and carrying 12 random amino acids fused to the N‐terminus of pIII. B) Phages recognizing PSI were identified by conversion of a fluorogenic substrate catalyzed by HRP. Afterward, the phages were amplified and sequenced to identify the binders against PSI. C) PSI oriented on a conducting surface with the help of selected short peptides carrying an anchoring group.

Afterward, the eluted phages were used to infect *Escherichia coli* to amplify single‐phage clones. The single clones were sequenced (see Table S1, Supporting Information) and their binding affinity toward PSI was tested. We prepared three different surfaces: the first contained PSI without any preferential orientation as utilized for the panning procedure; the second was coated with a monolayer of bovine serum albumin (BSA); and the third was decorated with PSI trimers oriented with the help of an anti‐PsaC antibody (which can orient PSI, but decouples it from the surface electronically) with the lumen side facing away from the surface and therefore exclusively accessible for binding (**Figure** **3**B: top, middle, and bottom panels, respectively). For biophotovoltaic and biofuel cell applications involving PSI, it is necessary to immobilize the complex such that it injects electrons or holes directly in the electrode beneath, but not both, otherwise the charges will recombine at the electrode's surface, bypassing the external circuit. We ensured such orientation as follows. We first employed an antibody against PsaC that harbors the F_B_ cluster—the final unit of the ETC where electron ejection takes place. When this antibody is immobilized, it forces the stromal side to orient toward the surface, preventing the binding of phages that recognize that part of PSI. We then performed binding studies to the three different surfaces (described above) in a modified phage‐(enzyme‐linked immunosorbent assay) (phage‐ELISA) by incubating the surfaces in a solution of single clones of M13 conjugated to HRP. After washing, we added the fluorogenic substrate and determined the relative amount of bound phages via the fluorescence intensity of the surface in real time. By comparing the signal from the randomly oriented PSI to those bound to PsaC antibodies, we could differentiate between peptide sequences that bind PSI indiscriminately versus those that selectively bind the desired stromal side. The outcome of this binding assay is shown in Figure 3A. The sequences 11 (P1, RDQNHYMYSARV), 12 (P2, IQAGKTEHLAPD), and 88 (P3, LATTSHMFMAKG) are the ones that bind PSI at the desired site. They show high affinity against the surface with PSI randomly oriented (i.e., with both sides available for binding), but they display low affinity against the pristine substrate and low affinity against the lumen side of PSI (the opposite side of anti‐PsaC antibody binding). Thus, they selectively bind on the stromal side (the same side as the anti‐PsaC antibody), as evidenced by the low fluorescence signal when the antibodies and surface shield this side.

**Figure 3:**
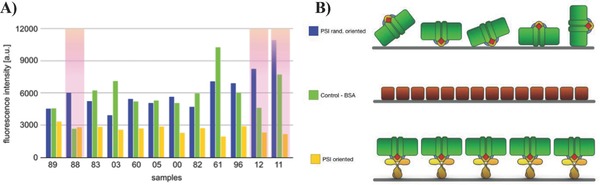
PD results. A) Fluorescence signal from the binding assay of single clones toward (1) randomly oriented PSI, (2) BSA, and (3) oriented PSI. B) Schematic representation of the different well's surfaces used to identify peptides binding the lumen side of PSI. Top: PSI is covalently linked without any preferential orientation. Middle: BSA‐coated well was used as control without PSI target. Bottom: PSI is linked to the well via antibodies. The specific interaction between the antibody and PsaC ensures that PSI exposes only its lumen side.

After identifying peptide sequences that bind PSI, we characterized the ability of these peptides to orient PSI on a conducting substrate by c‐AFM. Therefore, the peptide sequences were modified at the C‐terminus with a phosphorylated serine residue allowing binding to an ITO surface.^25^ For immobilization, PSI trimers were first incubated with the modified peptides and subsequently drop cast on the ITO substrates. For comparison, the ITO surface was functionalized with a small molecule, dihydroxyacetone phosphate (DHAP), which forms SAMs capable of biasing the subsequent self‐assembly of PSI toward one orientation as reported previously.^22^

To gain insights into the orientation of PSI, we performed c‐AFM studies on the three different peptide‐immobilized PSI layers and the DHAP directing linker as depicted in **Figure** **4**. The orientation of PSI on a conducting surface can be determined by c‐AFM, taking advantage of the tendency of the complexes to induce asymmetric *I–V* curves depending on their orientation. The origin of this rectification on Au substrates was recently elucidated and is most likely due to the permanent dipole of the PSI protein scaffold and not the cofactors of the ETC.^26^ When PSI is directed with the F_B_ side or PsaC toward the substrate (i.e., the bottom electrode), a higher electrical current is measured at negative bias compared to the electrical current at positive bias giving rise to *I–V* data that are asymmetric about zero. This is the same orientation afforded by the PD‐selected peptides. The same orientation was confirmed on ITO using DHAP as short linker molecule by comparing with data from biophotovoltaic devices incorporating PSI.^22^ In contrast, the opposite orientation, which is accessible with director SAMs of thiolates, gives rise to the opposite asymmetry. When PSI is lying with the axis of the ETC parallel to the surface (sideways) or if it is thermally denatured, current rectification is absent (i.e., the *I–V* data are symmetric). Guided by these observations, we analyzed the three different substrates with the three different peptides and an additional substrate bearing a scrambled sequence of peptide ALF (ALFHYNTHGSLH) acting as directors for the assembly of PSI and the short directing molecule DHAP. For convenience purposes, only the first three amino acids of each sequence will be used as identification.

**Figure 4:**
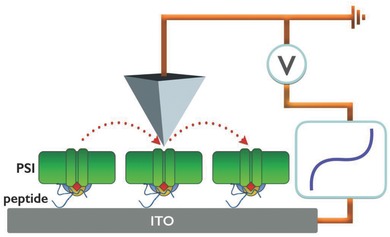
Schematic picture of c‐AFM setup. Studying the orientation of PSI trimers by c‐AFM oriented with the help of selected short peptides by the phage display technique.

We used Pt/Ir probes for c‐AFM studies in contact mode on a sample at zero voltage to record the topography of the PSI layers (Figure S2, Supporting Information). From these data we identified individual PSI trimers on the ITO surface as an approximately round, bright spot. We then set the c‐AFM to *I–V* ramping to record the electrical characteristics of individual PSI trimers, examining at least 100 trimers for each of the three peptide linkers. The resulting *I–V* curves show orientation‐dependent electrical behavior as described above. The expected, downward configuration of PSI shown in Figure 2C is preferred for all four peptides; however, as is shown in (**Table** **1**), only peptide IQA (P2) orients PSI almost exclusively in this configuration. The SAMs of PSI formed on the other three peptides also contained trimers in the parallel and (scarcely) upward configuration (*I–V* plots for IQA (P2) can be found in Figure S3, Supporting Information).

**Table 1:** PSI orientations controlled by short directing molecule and peptides

	Up [%]	Middle [%]	Down [%]
DHAP (short directing molecule)	7	13	80
LAT (P3)	10	13	77
RDQ (P1)	1	15	84
ALF (scramble)	5	9	86
IQA (P2)	1	1	98

After quantifying the orientation of PSI complexes one by one via c‐AFM, we investigated the macroscopic electrical properties of SAMs of PSI using the same four peptides and the short linker DHAP using EGaIn as a top electrode. In a recent study, SAMs of partially oriented PSI using two different short linkers showed orientation‐dependent electrical properties using EGaIn as a top electrode.^26^ Additionally, by measuring the charge transport through these two partially oriented PSI layers at temperatures between 198 and 298 K, it was shown that the mechanism of charge transport through PSI is most likely tunneling due to the lack of any apparent thermally activated processes. Following the same procedure (at room temperature), we compared the asymmetry (χ) of the *J/V* characteristics of the peptides (without PSI) and SAMs of PSI with the peptides with PSI complexes by computing the differences of ∆log|χ|. This value reflects the combined influence of the population of PSI trimer orientations—it gives insight into how the distribution of orientations and the assemblies of the underlying peptides influence devices as opposed to looking at isolated trimers. By comparing ∆log|χ| between the bare peptides and the SAMs of PSI on the peptides we can ascribe any observed asymmetry to either or both.

We fabricated test‐bed devices by incubating PSI with the modified peptides and subsequently drop‐casting the PSI‐peptide complexes onto clean ITO substrates. In comparison to macroscopic studies, the ITO surface was functionalized with an SAM of DHAP as described elsewhere.^22^ The EGaIn measurement protocol is described in detail separately.^26^ We contacted multiple PSI complexes on monolayers of different peptides—RDQ, ALF, LAT, and IQA—by lowering a conical tip of EGaIn^27^ (≈25 µm in diameter) and measuring conductance under dark conditions when PSI is not photoactivated. By biasing from −1 V to 1 V we were able to study the asymmetry parameter (χ), defined as χ = (*J* at −1 V)/(*J* at +1 V) according to the wiring of c‐AFM (which is backward from EGaIn).

**Figure** **5**A depicts (not to scale), the junction that is formed when the oxide layer contacts the biological complexes oriented by the peptides. Figure 5B shows the difference in log|χ| at 1 V of a monolayer of only modified peptides versus the peptides with the PSI complexes. Differences in log|χ| are an indication of the effect of the PSI/peptide versus possible asymmetry induced by the peptides, which are themselves highly oriented (otherwise they would not be able to orient PSI). Thus, we ascribe the observation of similar values of log|χ| to the low coverage of PSI complexes on RDQ and LAT because, due to the noncovalent nature of the peptide–PSI interaction and the strong affinity of the peptides for ITO, the PSI/peptide complex dissociated and the surface is passivated mainly by free peptide.^26^ That is, we expect ∆log|χ| to increase monotonically with orientation, but a lower surface coverage of PSI on LAT could lower ∆log|χ|.

**Figure 5:**
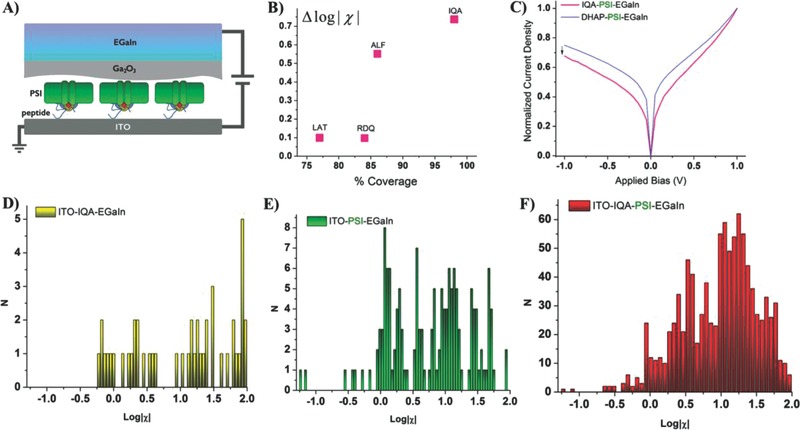
EGaIn characterizations. A) Schematic picture of the EGaIn junctions fabricated on PSI trimer monolayer. B) Difference in log|χ| of monolayers of only modified peptides versus the peptides with the PSI complexes. The percentage of oriented complexes was established by measuring the devices with c‐AFM and the value of *R* from *J/V* traces of large‐area measurements. C) Reference experiment of PSI trimers oriented by the short linker dihydroxyacetone phosphate, where the rectification was measured as log|χ| = 1.22. D) IQA peptide without PSI showed some rectification properties. E) PSI trimers exposed directly to nonmodified ITO surface showed a low rectification of log|χ| around 0. F) PSI trimers directed with IQA peptide showed the highest rectification as indicated by log|χ|.

The trend observable in our results is the increasing absolute value of ∆log|χ|. It is 0.097 for RDQ, 0.099 for LAT, 0.551 for ALF, and 0.737 for IQA (Figure 5B). This trend may be due to a stronger binding of PSI to the peptides or better affinity of the peptides to the substrate and therefore denser coverage of PSI (histograms of all traces can be found in Figure S4, Supporting Information).

Figure 5D–F shows the histograms at 1 V of devices comprising only IQA peptide (D), complexes deposited directly randomly onto ITO (E), and PSI oriented with the IQA peptide (F). EGaIn junctions comprising only IQA peptides were unstable, yielding mostly short‐circuit (ohmic) characteristics, indicating that peptide IQA itself is not as robust as PSI. The transport properties of bare ITO are quite sensitive to adsorbates that lack structure and as such, it was difficult to characterize (presumably) randomly oriented PSI on bare‐ITO, which exhibited a broad distribution of χ‐values.^28^ By contrast, of all of the substrates tested, PSI oriented with IQA peptide was the most stable and reproducible. In addition to experiments on the PD peptides, we measured SAMs of PSI with the short directing linker DHAP. These devices exhibited a value of log|χ| = 1.22, which is close to those of PSI oriented with peptides, generally, but still lower than PSI oriented with IQA peptide (Figure 5C).

After having thoroughly characterized the ability of IQA to orient PSI and examined the effects of orientation in single complexes and large areas, we integrated oriented SAMs of PSI into bioelectronic devices. We chose BHJ solar cells containing PSI as electrode modifiers because these devices may act as a biophysical tool to determine the influence of the orientation of PSI in functional devices under illumination.^22^ In this way, we extend the characterization of the near‐perfect orientation of SAMs of PSI by peptides selected by PD and demonstrate that they retain their function/orientation in standard sandwich‐device architectures.

The magnitude of *V*_OC_ in an organic BHJ solar cell is sensitive to the difference in work function of the electrodes, which can be modified with interfacial layers containing oriented dipoles.^29^, ^30^ When a PSI monolayer is illuminated, electrons are directed to the acceptor side (F_B_–Fe_4_S_4_ cluster) while positive charges (holes) remain at the P700 donor site. As a consequence of charge separation, a dipole between the semiconductor electrode and the organic BHJ is created, which effectively modifies the work function of the electrode.^31^ (This is a different phenomenon from the effects of embedded dipoles on c‐AFM and EGaIn junctions, which were measured in the dark.) Since *V*_OC_ is sensitive to the work function difference between the bottom and the top electrode, the dipole orientation can be determined from the shift in work function. We chose the same material, ITO, as an electrode to allow direct comparison with the c‐AFM and EGaIn results. We immobilized the PSI monolayer as described above. Additionally, we immobilized PSI with DHAP, which directs the majority of PSI trimers with the F_B_ site downward to the surface (same orientation as peptides) as a control. We formed the active layer by spin coating a 1:4 mixtures (by wt%) of the p‐type conjugated polymer poly[2‐methoxy‐5‐(2‐ethylhexyloxy)‐1,4‐phenylenevinylene] (MEH‐PPV) and the fullerene derivative phenyl‐C61‐butyric acid methyl ester (PCBM) in chlorobenzene on the SAMs of PSI. As the last step of device fabrication, we deposited either electron‐extracting (LiF/Al) or hole‐extracting (MoO_3_/Al) electrodes atop the MEH‐PPV:PCBM/PSI/peptide/ITO stack by thermal evaporation to produce normal and inverted photovoltaic devices, respectively (**Figure** **6**A,B).

**Figure 6:**
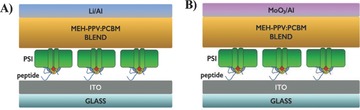
BHJ solar cell devices. Architectures of fabricated conventional A) solar cells with LiF/Al top electrode and B) inverted cells with MoO_3_/Al top electrode.

Normal and inverted BHJ devices are differentiated by the direction of photogenerated current. In both configurations, current is mainly generated in the 120 nm thick organic BHJ, which absorbs substantially more light than the PSI monolayer. As a consequence, the PSI layer acts as a modifier of the electrodes, providing indirect information about the net orientation of the PSI complexes. It should be noted that the photogenerated current by PSI is small compared to the current generated by MEH‐PPV:PCBM and that therefore the incorporation of PSI has only a minor influence on the total photogenerated current. The effect on the voltage, is, however, clearly visible. As shown in Table S2 (Supporting Information), *V*_OC_ decreases upon introducing the PSI layer in the device with the low work function LiF/Al top electrode. In contrast, when the PSI layer is integrated into the inverted cells (with the high work function MoO_3_/Al top electrode), *V*_OC_ increases. This behavior implies that the PSI monolayer shifts the vacuum level, lowering the work function of ITO, confirming that a sufficiently large fraction of the dipoles in the PSI layer are directed with F_B_ toward ITO (**Figure** **7**A). In this orientation, the photogenerated electrons of PSI are predominantly injected into the metal oxide. Moreover, there are significant differences between the peptides P1–P3 and scrambled ALF peptide compared to the small directing molecule DHAP.^22^ For the normal cells (with the low work function LiF/Al top electrode) incorporating DHAP, Δ*V*_OC_ was 0.03 V, while for all directing peptides a Δ*V*_OC_ greater than 0.1 V was obtained (Table S2, Supporting Information). These results clearly indicate that all peptides direct a larger fraction of PSI downward with F_B_ site being arranged in close proximity to the semiconductor and not to the organic material compared to the directing layer consisting of the small molecule. Similar results were obtained for the inverted solar cell configuration containing MoO_3_/Al top electrode (Figure 7B). There again, all of the peptides resulted in a larger Δ*V*_OC_ than for DHAP and IQA yielded the largest value of Δ*V*_OC_. The BHJ solar cell experiments undoubtedly prove the downward orientation of PSI by the selected peptides under illumination and, therefore, the orientation determined in the dark by c‐AFM and EGaIn experiments is retained in assembled, thin‐film photovoltaic devices.

**Figure 7:**
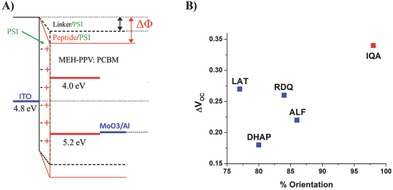
PSI induced Δ*V*_OC_ changes. A) Energy diagrams of fabricated solar cells with top MoO_3_/Al electrode. Immobilized PSI monolayer via directing peptides induces change in work function of cathode electrode (Δ*Φ*), due to own dipole moment. B) Δ*V*_OC_ difference measured between fabricated BHJ solar cells containing PSI monolayers directed with corresponding peptides versus the orientation measured with c‐AFM.

The orientation of photoactive proteins such as PSI on a surface is of major importance either to study their biophysical behavior or to exploit their superb light‐induced charge separation capabilities in bioinspired solar and fuel cells. We used phage display to identify binders against the photosynthetic complex PSI. The selection strategy was designed in such a way that only one round of selection was necessary to obtain binders and that these binders are directed to a desired side within this megadalton multiprotein complex, i.e., the stromal side (where electrons are ejected after photoexcitation). Three 12 mer sequences, obtained by phage display, enabled the immobilization of PSI with the majority of complexes oriented with the opposite face toward a semiconductor surface after the peptides are modified with an appropriate surface anchoring group. One of the peptides was able to direct almost all PSI molecules exclusively in one orientation. It is crucial that the orientation and its effects be characterized rigorously in the dark, over large areas and in the light and that the orientation survive processing into thin‐film devices. Therefore, we characterized single PSI complex and SAMs and incorporated PSI‐modified ITO into photovoltaic devices.^22^ We determined the orientation of single complexes by conducting AFM, large‐area junctions with EGaIn, and under illumination in photovoltaic devices. In all three cases, the collective action of the dipoles intrinsic to the complexes (dark) or the electron‐transport chain (illumination) affected charge‐transport systematically as a function of the direction/degree of orientation.^26^ Future work will be directed toward exploiting these peptides for integrating PSI in other biosolar and biofuel cells and to functionalize PSI in a mild, noncovalent fashion, which requires only the addition of functionalized peptides and their binding to PSI without any chemical coupling reagent.^32^ Finally, this work showcases that the phage display technology can be exploited to immobilize and orient protein targets in bioelectronic devices.

## Supporting information

As a service to our authors and readers, this journal provides supporting information supplied by the authors. Such materials are peer reviewed and may be re‐organized for online delivery, but are not copy‐edited or typeset. Technical support issues arising from supporting information (other than missing files) should be addressed to the authors.

Supplementary
