# Supplementary material for: Orientation and Incorporation of Photosystem I in Bioelectronics Devices Enabled by Phage Display
Source: Adv Sci (Weinh). 2017 Jan 31;4(5):1600393. doi: 10.1002/advs.201600393 (PMC5441502; doi:10.1002/advs.201600393)
Supplement: Supplementary file 1 — Supplementary [file ADVS-4-na-s001.pdf]

Copyright WILEY-VCH Verlag GmbH & Co. KGaA, 69469 Weinheim, Germany, 2017.

Supporting Information

for *Adv. Sci.,* DOI: 10.1002/advs.201600393

Orientation and Incorporation of Photosystem I in Bioelectronics Devices Enabled by Phage Display

Pavlo Gordiichuk, Diego Pesce, Olga E. Castañeda Ocampo, Alessio Marcozzi, Gert-Jan A. H. Wetzelaer, Avishek Paul, Mark Loznik, Ekaterina Gloukhikh, Shachar Richter, Ryan C. Chiechi, and Andreas Herrmann**

DOI: 10.1002/advs.201600393
Article type: Communication

Supporting Information

Orientation and Incorporation of Photosystem I in Bioelectronics Devices Enabled by Phage Display

Pavlo Gordiichuk, Diego Pesce, Olga E. Castañeda Ocampo, Alessio Marcozzi, Gert-Jan A. H. Wetzelaer, Avishek Paul, Mark Loznik, Ekaterina Gloukhikh, Shachar Richter, Ryan C. Chiechi, Andreas Herrmann

Cell culture and thylakoid membrane preparation

The thermophilic cyanobacterium T. elongatus BP1 was grown under agitation (150 rpm) in BG11 medium. The temperature was kept at 56°C, continuous light applied at 50-60µEinstein x m-2 x s-1 and cell growth pursued until late log phase. At last, the cells were harvested by centrifugation [JLA 9.100 rotor, Beckman; 7500xg; 15min], re-suspended in Buffer A [20mM HEPES (pH7.5); 10mM MgCl2; 10mM CaCl2; 500mM Mannitol], snap-frozen in liquid nitrogen and stored at -80°C.

Thylakoid membranes were prepared according to the following protocol, which represents a combination of two previously described preparation methods. To this end, fresh or frozen cells were resuspended in Buffer A and homogenized five times using a Dounce homogenizer. After addition of lysozyme [final concentration: 0,5% (wt/vol)] and a tip of a spatula of DNase, the cell suspension was incubated under slow agitation for 45min at 37°C in the dark. Subsequently, the cells were lysed by two passages through a French Press (15000psi; Constant Systems Limited, UK). Membranes were collected by centrifugation [JLA 16.250 rotor, Beckman; 38000xg; 20min] and washed with Buffer A containing 3M NaBr.

Afterwards, the membrane suspension was washed once with Buffer A and three times with Buffer containing 0,05% DDM (n-dodecyl-β-D-maltoside) in order to remove the phycobilisomes. Finally, the thylakoid membranes were solubilized by incubation in Buffer A supplemented with 0,6% DDM for 30min at 20°C in the dark. Non-solubilized material was pelleted by centrifugation [JLA 16.250 rotor, Beckman; 16000rpm (38000xg); 20min] and the supernatant subjected to subsequent purification steps.

Photosystem I purification

For PSI purification, fast liquid protein chromatography was applied on solubilized thylakoid membranes. The chromatographic purification was performed on a ÄKTAexplorer [GE Healthcare] using an anion exchange column [HiTrapTM Q HP, GE Healthcare]. After column equilibration with Buffer A + 0,03% DDM, the sample was applied and subsequently eluted by a linear gradient of [0-1M] $MgSO_4$. The green fluorescent fractions were collected and desalted with Buffer A + 0,03% DDM using Vivaspin 20 columns [molecular weight cut-off: 100kDa; GE Healthcare]. Finally, the purified PSI sample was adjusted to a Chl a concentration of 800 µM [with Buffer A + 0,03% DDM], snap-frozen in liquid nitrogen and stored at -80°C. Determination of Chlorophyll a and Protein Concentration: Chl a determination was performed in 100% methanol as described in Porra et al. [1]

Absorption and Fluorescence Spectra: All absorption and fluorescence spectra were recorded with a SpectraMax M2 spectrophotometer (Molecular Devices, US) at 25°C.

Phage Display

Chemical coupling of horse radish peroxidase (HRP) to M13 phage library: Coupling of HRP (EZ-Link TM Plus Activated Peroxidase - Thermo scientific) was performed following the vendor’s protocol using 10e11 phages (NEB #E8110S) and 1mg of peroxidase. HRP was applied in excess in relation to the phage to ensure high degree of functionalization of the phage particles. The HRP, which was activated by periodate treatment at the sugar moieties to exhibit aldehyde functions, which are expected to react with amino groups on the phage

surface of the pVIII coat protein. The Schiff base formation was carried out in sodium carbonate pH 9.8 at 16°C for 16 h in a shaker at 700 rpm. Then, reductive amination was induced by addition of 10 µl of sodium cyanoborohydride (5 M) to the reaction mixture in order to stabilize the conjugation of HRP to M13 coat proteins. After terminating the reaction by addition of ethanolamine 3 M pH 10, the excess of unreacted HRP was separated from the phages by filtration using a 50.000 Da cutoff spin column. The flow-through (i.e. the HRP) was further tested for nonspecific adsorption to the functionalized plates to verify any level of background signal due to unreacted HRP in the biopanning experiment. No background noise was detected (data not shown).

Both the supernatant (containing the phages conjugated to HRP) and the flow-through (containing quenched HRP) were tested for infectivity and peroxidase activity. As expected, only the phage-containing fraction was infective and both fractions showed high HRP activity. These results prove that the phage particles conjugated to peroxidase are still infective and HRP is still catalytically active, two criteria essential for our selection system.

Single round phage display (PD). A single round PD selection was carried out using HRP-conjugated phage particles. The original library of phages expressing 12mer peptides at the N-terminus of their pIII protein was purchased from NEB (NEB #E8110S). After HRP-conjugation, a population of 10^9 phages per well was selected to be exposed to PSI-modified surfaces. The wells of the plate were prepared according to the manufacturer's instruction (Nunc™ Amino™ Immobilizer Surface, Thermo Scientific). In brief, the wells were incubated with 100 µl of a solution of PSI (300 µM) with gentle agitation (200 rpm) at RT over-night. The wells were then aspirated and washed three times with PBST containing 1% BSA (w/v).

The buffers used for the incubation, wash and elution steps are respectively: PBS pH 7.0, PBST (PBS + 0.1% Tween 20) and Tris-glycine pH 2.2.

In previous experiments, we observed some binding affinity of the phage library to the pristine plate, probably due to plastic binders being present in the library. Therefore, an initial negative selection against blank wells was performed. Next, the phages were split in two sub populations and incubated separately in wells with and without PSI target. After extensive washing steps using PBST, peroxidase substrate (QuantaBlue Fluorogenic Peroxidase Substrate Kit-ThermoFisher #15169) was added to each well to determine the presence of bound phages. When a signal from the blank wells was absent and a clear signal originating from the PSI containing wells was detected, elution with Tris-glycine buffer was initiated. Following the same procedure, the elution step was monitored to prevent phages remaining bound to the target. Before and after elution, the developing buffer (QuantaBlu™ Fluorogenic Peroxidase Substrate Kit) for the HRP was added to the wells and the activity of peroxidase was measured over time in RFU.

Conducting AFM measurements.

The height images of PSI were measured in tapping mode with TESP-V2 probes by a MultiMode 8 AFM Microscope with System Controller V. External TUNA mode was used for the height and electrical *I-V* characteristics recording in contact mode. ANSCM-PC probes with a spring constant of k = 0.2 N/m, tip radius 30 nm and tip height 2.5-8 µm were utilized. The scanning region was selected with a size of up to 2 µm by 2 µm. The scanning rate and number of lines were selected to be 0.2 Hz and 512 lines/sample, respectively. For each image and electrical characterizations, a new probe was employed to avoid AFM tip broadening due to wear and contaminations from the measuring surface. The AFM images were analyzed with NanoScopeAnalysis software.

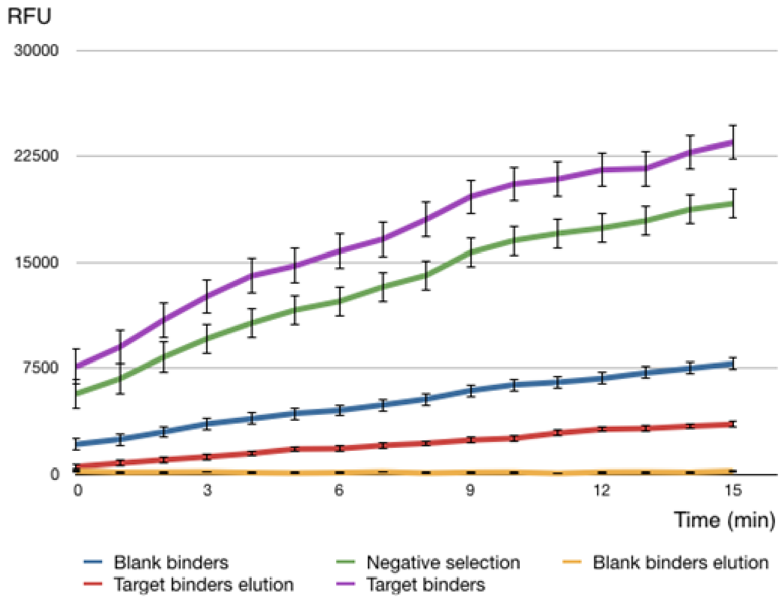

Fig. S1. Readout of the phage display against PSI carried out with HRP conjugated M13 PhD 12. Signal of the phages bound to the negative control wells after incubation (green line). Unbound phages were transferred in wells with and without PSI and washed (violet and blue lines, respectively). The yellow and red lines represent the signal from blank well and PSI after elution. Fluorescence intensities are the average of three measurements with corresponding error bars.

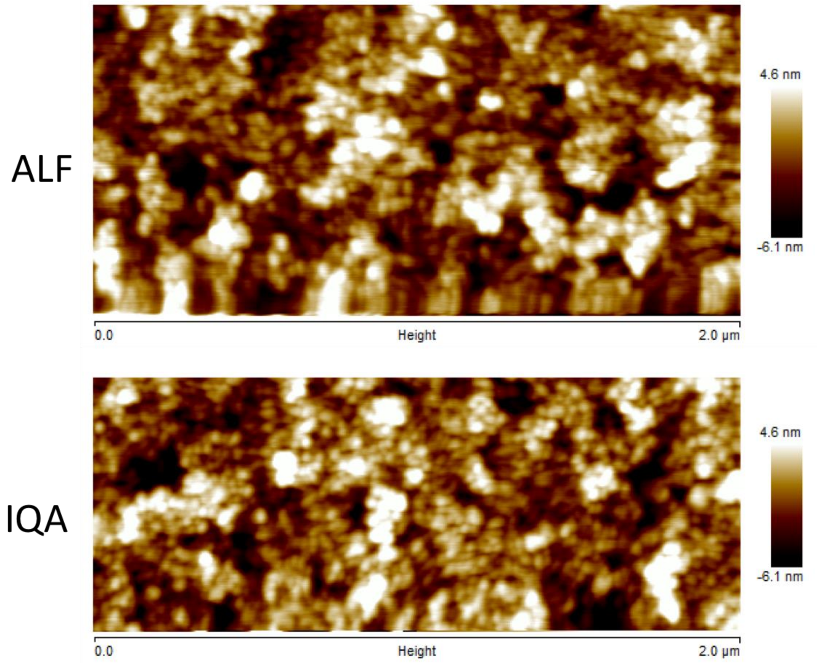

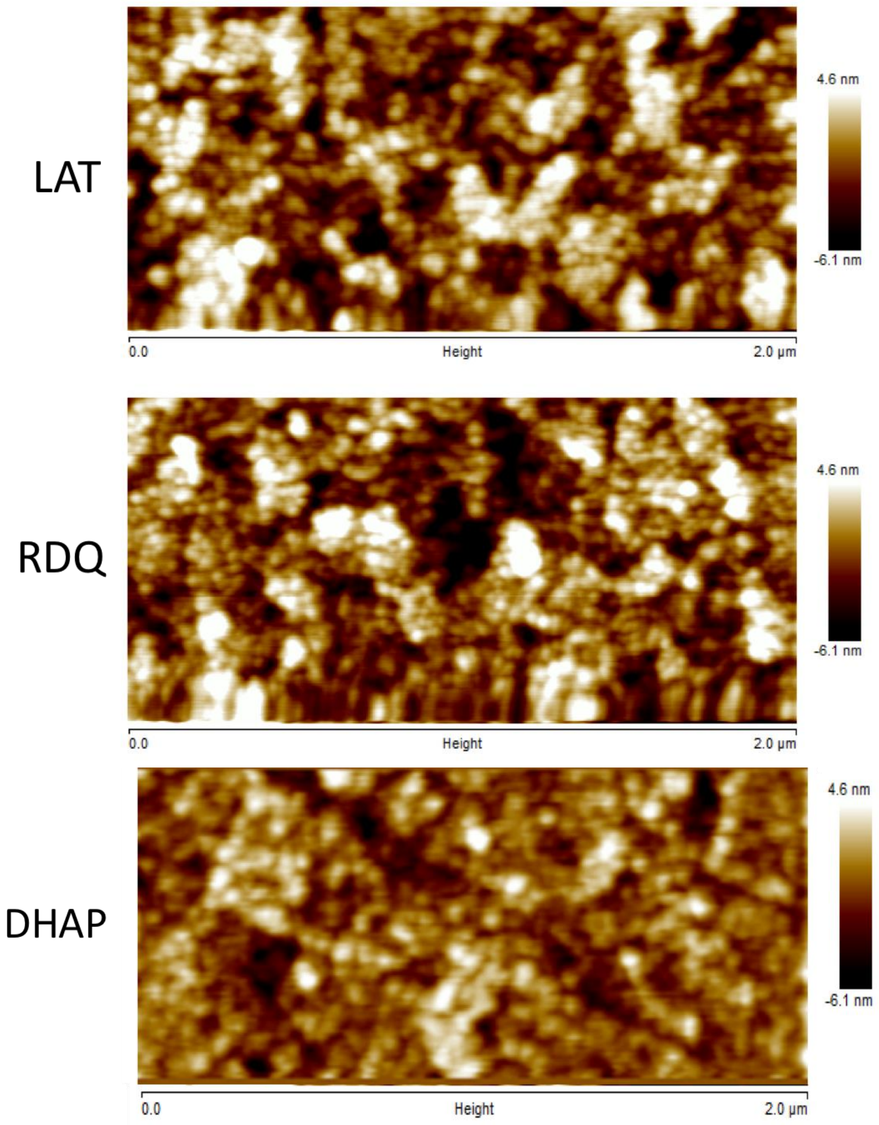

Fig. S2. Topographical images of the PSI monolayers immobilized with the help of ALF, IQA, LAT, RDQ and DHAP linkers. Images were recorded in contact mode with the help of conducting Pt/Ir probe at force below 1 nN and zero applied electrical bias.

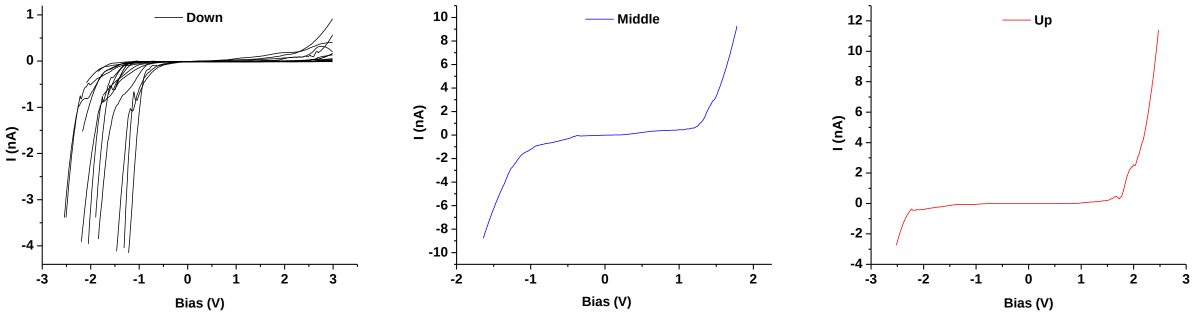

Fig. S3. C-AFM *I-Vs* on PSI oriented with IQA peptide. 15 Characteristic *I-V* plots are shown for PSI oriented down with the F_B cluster (left), one I-V for PSI oriented parallel to the surface (middle) and one for the case when PSI is oriented up (right).

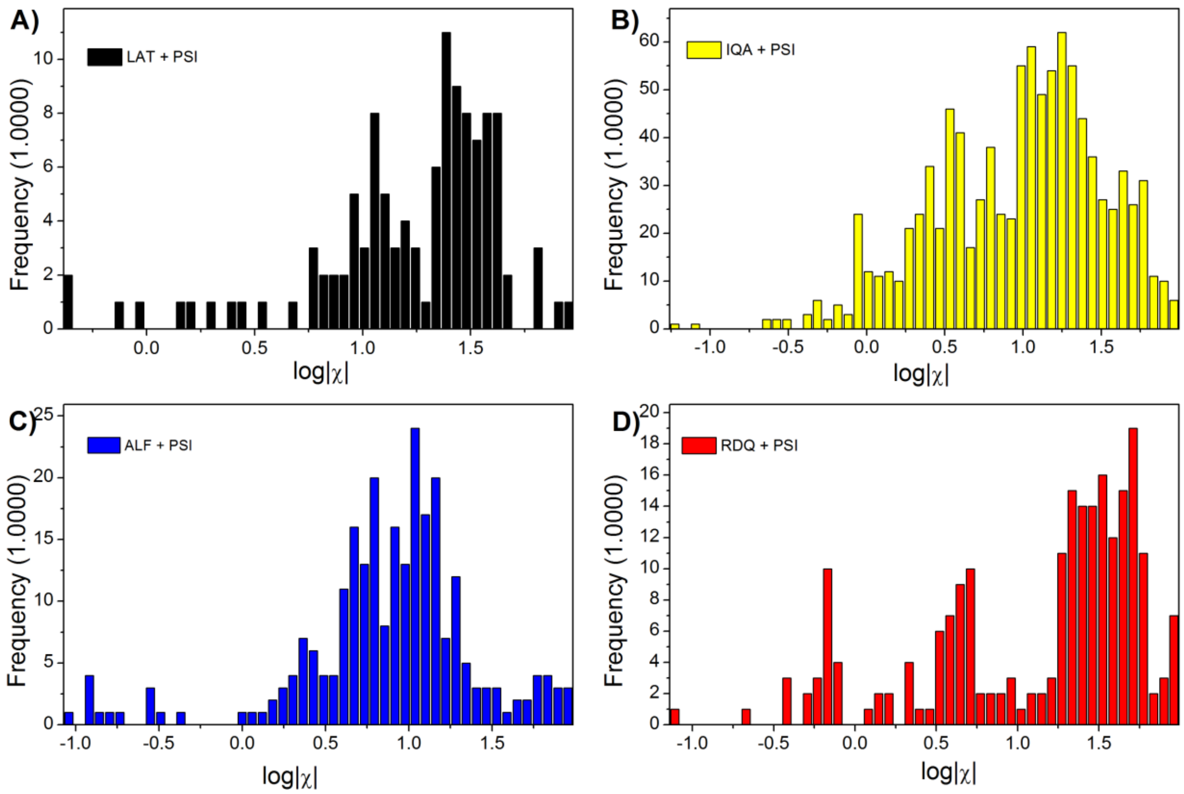

Fig. S4. Histograms of log|χ| recorded on PSI trimers oriented on ITO with different peptides as binders. (**A**) Is the histogram of the traces with LAT as orienting peptide which reached a percentage of 80%. (**B**) Is the histogram of PSI on IQA, the peptide that orients PSI in 98% of the cases. (**C**) represents PSI on ALF which orients 86% of the complexes and (**D**) is the peptide RDQ with 84% orientation.

Table S1. Amino acid sequences of the peptides selected by phage display.

Sequence ID	Sequence aa (N- to C terminus)
**89**	IPENSYSKTKYL
**88**	LATTSHMFMAKG
**83**	GSLMADSSVVAR
**03**	TNGYNTTMYAYR
**60**	GNNNMNPLTSND
**05**	DILDKSPQGKTH
**00**	YSHFHQMSAKSH
**82**	KNQLVLPSADQL
**61**	SVLNKHSTYVTS
**96**	HFTHGHYANLLS
**12**	IQAGKTEHLAPD
**11**	RDQNHYMYSARV

Table S2. Parameters of PSI trimer peptide solar cells.

Bottom electrode	Top electrode	Voc (V)	Jsc (A/m^2)	FF	ΔVoc (V)
**ITO**	LiF/Al	0.44±0.07	29.03±0.60	0.32±0.02	
**ITO/OH-linker/PSI**	LiF/Al	0.42±0.05	28.45±1.78	0.42±0.06	-0.03
**ITO/P3(LAT)/PSI**	LiF/Al	0.34±0.02	27.98±1.16	0.46±0.02	-0.1
**ITO/P1(RDQ)/PSI**	LiF/Al	0.29±0.02	20.74±0.93	0.46±0.03	-0.15
**ITO/P2(IQA)/PSI**	LiF/Al	0.29±0.04	21.41±2.66	0.47±0.02	-0.15
***ITO/(ALF)/PSI**	LiF/Al	0.26±0.03	20.19±1.25	0.43±0.02	-0.18
**ITO**	MoO3/Al	0.21±0.03	22.83±1.00	0.46±0.07	
**ITO/OH-linker/PSI**	MoO3/Al	0.39±0.01	21.8±0.55	0.46±0.08	0.18
**ITO/P3(LAT)/PSI**	MoO3/Al	0.46±0.01	27.13±0.48	0.46±0.08	0.25
**ITO/P1(RDQ)/PSI**	MoO3/Al	0.41±0.01	27.49±0.75	0.46±0.10	0.2
**ITO/P2(IQA)/PSI**	MoO3/Al	0.48±0.02	28.81±0.95	0.46±0.11	0.27
***ITO/(ALF)/PSI**	MoO3/Al	0.42±0.01	28.17±1.26	0.46±0.12	0.21

peptide (ALF) is a scrambled peptide.

[1] Porra R. J.; Thompson W. A.; Kriedemann P. E. Determination of Accurate Extinction Coefficients and Simultaneous-Equations for Assaying Chlorophyll-a and Chlorophyll-B Extracted with 4 Different Solvents - Verification of the Concentration of Chlorophyll Standards by Atomic-Absorption Spectroscopy. *Biochim. Biophys. Acta* **1989**, 975, 384.
